# Supplementary material for: Resolving the full spectrum of human genome variation using Linked-Reads
Source: Genome Res. 2019 Apr;29(4):635–45. doi: 10.1101/gr.234443.118 (PMC6442396; doi:10.1101/gr.234443.118)
Supplement: Supplemental Material [file supp_gr.234443.118_Supplemental_longranger-2.2.zip › longranger-2.2.tar.bz2!/lr2.2-public/lib/stan/cmdstan/stan/doc/api/html/do__bfgs__optimize_8hpp.html]

Stan: src/stan/common/do\_bfgs\_optimize.hpp File Reference


|  |  |
| --- | --- |
|  | Stan  2.5.0  probability, sampling & optimization |


- Main Page
- Related Pages
- Namespaces
- Classes
- Files

- File List
- File Members

All Classes Namespaces Files Functions Variables Typedefs Enumerations Enumerator Friends Macros Pages

- src
- stan
- common

Namespaces |
Functions

do\_bfgs\_optimize.hpp File Reference

`#include <fstream>`  
`#include <iostream>`  
`#include <iomanip>`  
`#include <vector>`  
`#include <stan/gm/error_codes.hpp>`  
`#include <stan/common/do_print.hpp>`  
`#include <stan/common/write_iteration_csv.hpp>`  
`#include <stan/common/write_iteration.hpp>`

Go to the source code of this file.

|  |  |
| --- | --- |
| Namespaces | |
|  | stan |
|  | Probability, optimization and sampling library. |
|  | |
|  | stan::common |
|  | |

|  |  |
| --- | --- |
| Functions | |
| template<typename ModelT , typename BFGSOptimizerT , typename RNGT , typename StartIterationCallback > | |
| int | stan::common::do\_bfgs\_optimize (ModelT &model, BFGSOptimizerT &bfgs, RNGT &base\_rng, double &lp, std::vector< double > &cont\_vector, std::vector< int > &disc\_vector, std::fstream \*output\_stream, std::ostream \*notice\_stream, bool save\_iterations, int refresh, StartIterationCallback &callback) |
|  | |


---

[ Stan Home Page ]

*© 2011–2014,
Stan Development Team.*
